# Biological Phosphorus Removal During High-Rate, Low-Temperature, Anaerobic Digestion of Wastewater

**DOI:** 10.3389/fmicb.2016.00226

**Published:** 2016-03-03

**Authors:** Ciara Keating, Jason P. Chin, Dermot Hughes, Panagiotis Manesiotis, Denise Cysneiros, Therese Mahony, Cindy J. Smith, John W. McGrath, Vincent O’Flaherty

**Affiliations:** ^1^Microbial Ecology Laboratory, Microbiology, School of Natural Sciences and Ryan Institute, National University of Ireland GalwayIreland; ^2^School of Biological Sciences and the Institute for Global Food Security, The Queen’s University of BelfastBelfast, UK; ^3^School of Chemistry and Chemical Engineering, The Queen’s University of BelfastBelfast, UK

**Keywords:** sewage, LtAD, microbial ecology and physiology, phosphate removal, hybrid reactor, psychrophilic

## Abstract

We report, for the first time, extensive biologically mediated phosphate removal from wastewater during high-rate anaerobic digestion (AD). A hybrid sludge bed/fixed-film (packed pumice stone) reactor was employed for low-temperature (12°C) anaerobic treatment of synthetic sewage wastewater. Successful phosphate removal from the wastewater (up to 78% of influent phosphate) was observed, mediated by biofilms in the reactor. Scanning electron microscopy and energy dispersive X-ray analysis revealed the accumulation of elemental phosphorus (∼2%) within the sludge bed and fixed-film biofilms. 4′, 6-diamidino-2-phenylindole (DAPI) staining indicated phosphorus accumulation was biological in nature and mediated through the formation of intracellular inorganic polyphosphate (polyP) granules within these biofilms. DAPI staining further indicated that polyP accumulation was rarely associated with free cells. Efficient and consistent chemical oxygen demand (COD) removal was recorded, throughout the 732-day trial, at applied organic loading rates between 0.4 and 1.5 kg COD m^-3^ d^-1^ and hydraulic retention times of 8–24 h, while phosphate removal efficiency ranged from 28 to 78% on average per phase. Analysis of protein hydrolysis kinetics and the methanogenic activity profiles of the biomass revealed the development, at 12°C, of active hydrolytic and methanogenic populations. Temporal microbial changes were monitored using Illumina MiSeq analysis of bacterial and archaeal 16S rRNA gene sequences. The dominant bacterial phyla present in the biomass at the conclusion of the trial were the Proteobacteria and Firmicutes and the dominant archaeal genus was *Methanosaeta*. *Trichococcus* and *Flavobacterium* populations, previously associated with low temperature protein degradation, developed in the reactor biomass. The presence of previously characterized polyphosphate accumulating organisms (PAOs) such as Rhodocyclus, Chromatiales, Actinobacter, and Acinetobacter was recorded at low numbers. However, it is unknown as yet if these were responsible for the luxury polyP uptake observed in this system. The possibility of efficient phosphate removal and recovery from wastewater during AD would represent a major advance in the scope for widespread application of anaerobic wastewater treatment technologies.

## Introduction

High-rate anaerobic digestion (AD) wastewater treatment technologies provide low-cost and effective removal of pollutants, with many advantages over other catalytic processes, combined with the recovery of energy in the form of methane. Despite many instances of its successful application, a drawback associated with AD wastewater treatment has been the inability to achieve acceptable – even moderate – levels of inorganic nutrient, in particular phosphate (P), removal that would avoid the need for extensive aerobic biological or chemical post-treatment (McGrath and Quinn, 2003; Caravelli et al., 2012; Hauduc et al., 2015).

Phosphate recovery and re-use from various sources is urgently needed to address an imminent P availability crisis. The current practice of mining rock-P (an exhaustible resource) for agricultural use is unsustainable. Wastewater streams offer an important opportunity to recover and recycle P, thus helping to close the P cycle. Indeed, up to 30% of world demand for P could theoretically be satisfied by its recovery from domestic waste streams alone (Gilbert, 2009). To date, the well-established Enhanced Biological Phosphate Removal (EBPR) systems have been applied for P removal from wastewaters. EBPR is based upon the exposure of activated sludge to alternating anaerobic and aerobic phases: P removal across the system is achieved via the intracellular accumulation of polyphosphate (polyP) by specialized group (or groups) of microorganisms. However, in reality these systems can demonstrate variability in performance, as polyP uptake is dependent on a number of operational and microbiological conditions that remain to be fully elucidated (McGrath and Quinn, 2003; Zeng et al., 2013; Yu et al., 2014; Motlagh et al., 2015). To date, biological P removal and recovery during AD wastewater treatment had not been reported. Despite this, luxury polyP uptake has been observed in strictly anaerobic archaeal species (Rudnick et al., 1990; Smirnov et al., 2002; Auernik et al., 2008; Toso et al., 2011; Orell et al., 2012) indicating the possibility for application of polyP synthesis as a means for P removal under anaerobic conditions. The potential for efficient removal and recovery of P during AD of dilute wastewaters was significantly advanced by Hughes et al. (2011) using a novel hybrid bioreactor system incorporating a fixed-film section of packed pumice stone. Despite this advance, the precise mechanism of phosphate removal and the role of the microbial consortia within the system remain to be ascertained.

Sewage wastewater is an important source of P into the environment, which if not intercepted, can lead to eutrophication of receiving water bodies (Khan et al., 2011; Barca et al., 2012; Wang and Pei, 2013). Sewage is generally treated in developed countries using aerobic biological systems, such as the activated sludge process, although these do not rank well in terms of sustainability criteria due, for example, to the high levels of energy required for aeration. Anaerobic treatment technologies have been successfully implemented for low-strength domestic sewage, but mainly in tropical/warm temperature regions (Smith et al., 2012). In temperate climates, the requirement to heat wastewaters to facilitate mesophilic operation would negate any energy savings gained. Low temperature (psychrophilic) AD allows for the economically efficient application of AD to low-strength wastewaters in temperate regions (McKeown et al., 2012). A reduction in hydrolysis rates corresponding with the accumulation of biodegradable solids in high-rate reactor systems operated at short hydraulic retention times (HRTs), however, may preclude low-temperature anaerobic treatment opportunities. The capacity for, and the extent of, the development of efficient hydrolytic biomass remain largely unexplored. It is known and well-studied, however, that increased methanogenic and acetogenic activity can develop during low-temperature AD, driven both by shifts in the microbial community and the development of psychrotolerance in organisms, such as *Methanosaeta* (McKeown et al., 2012; Zhang et al., 2012; Gunnigle et al., 2015). Some recent studies (Regueiro et al., 2014; Gunnigle et al., 2015) indicate that the Bacteroidetes and Proteobacteria phyla may be important in the case of a low-temperature shock. However, limited information is available on the bacterial community during prolonged low-temperature operation. Moreover, the roles played by the diverse bacterial species responsible for hydrolysis and acidification is only understood at a very basic level in AD generally. The capacity for enhanced hydrolysis, acidification and methanogenesis at low temperatures could underpin successful operation of future high-rate AD sewage treatment systems in temperate regions.

Our hypotheses were that high levels of P removal during AD would be achievable during high-rate treatment of synthetic sewage at low-temperature, in conjunction with highly efficient, low-temperature methanogenic biodegradation of the solid, colloidal and soluble fractions of a sewage wastewater: (i) at loading rates >1 kg total COD m^-3^ day^-1^; (ii) while producing an eﬄuent quality of <125 mg total COD l^-1^; (iii) with microbial community development resulting in increased hydrolytic, acidogenic, and methanogenic activity at low-temperatures. Our aim was to test these hypotheses in a laboratory-scale bioreactor system.

## Materials and Methods

### Reactor Design, Set-Up, and Operation

This study employed a glass laboratory-scale hybrid sludge bed/fixed-film (packed pumice stone) reactor (2.8 l working volume) as described by Hughes et al. (2011). The reactor was seeded with 20 g VSS l^-1^ of seed biomass [obtained through a sludge-screening step (Keating et al., 2012)]. The substrate used was a synthetic sewage based wastewater (SYNTHES) from Aiyuk and Verstraete (2004) at 500 mg l^-1^ COD_Tot_ outlined in **Table 1**. The reactor was operated at 12°C in a trial of 732 days. The trial was divided into five phases, each involving a different applied HRT and organic loading rate (OLR; **Table 2**) with Phase 4B marking a change in the fixed-film filter unit.

**Table 1:** Composition of 500 mg l^-1^ COD_Tot_ SYNTHES (Aiyuk and Verstraete, 2004).

Chemical components	Food ingredients	Trace metals
Urea (100 mg l^-1^)	Starch (131.2 mg l^-1^)	Cr(NO_3_)_3_-9H_2_O (0.9 mg l^-1^)
NH_4_Cl (12.5 mg l^-1^)	Milk powder (125 mg l^-1^)	CuCl_2_-2H_2_O (0.6 mg l^-1^)
Na-Acetate-3H_2_O (140.6 mg l^-1^)	Dried yeast (56.2 mg l^-1^)	MnSO_4_-H_2_O (0.1 mg l^-1^)
Peptone (18.7 mg l^-1^)	Soy Oil (31.2 mg l^-1^)	NiSO_4_-6H_2_O (0.3 mg l^-1^)
MgHPO_4_-3H_2_O (31.2 mg l^-1^)		PbCl_2_ (0.1 mg l^-1^)
K_2_HPO_4_-3H_2_O (25 mg l^-1^)		ZnCl_2_ (0.3 mg l^-1^)
FeSO_4_-7H_2_O (6.2 mg l^-1^)		
CaCl_2_ (6.2 mg l^-1^)		

**Table 2:** Reactor operation phases and associated operational conditions.

PHASE DAYS	Start-Up^∗^ 1–35	1 36–105	2 106–209	3 210–307	4A 308–487	4B 487–638	5 639–732
HRT^i^	36	36	24	18	12	12	8
TEMP^ii^	12	12	12	12	12	12	12
OLR^iii^	0.3	0.3	0.5	0.6	1	1	1.5
VLR^iv^	0.67	0.67	1.00	1.33	2	2	3
SLR^v^	0.03	0.03	0.05	0.10	0.16	0.16	0.23
SLR^vi^	0.02	0.02	0.03	0.05	0.08	0.08	0.11
UV^vii^	2.5	2.5	2.5	2.5	2.5	2.5	2.5

*^i^Hydraulic retention time (h); ^ii^Temperature (°C); ^iii^Organic loading rate (kg COD m**^-^**^3^ d**^-^**^1∗^; ^iv^Volumetric loading rate (m^3^ Wastewater m^-3^ Reactor d^-1^); ^v^Sludge loading rate based on granular sludge bed VSS estimated at each phase (kg COD kg [VSS]**^-^**^1^ d**^-^**^1^)^∗^; ^vi^Sludge loading rate (m^3^ Wastewater kg [VSS]**^-^**^1^ d**^-^**^1^); ^vii^Up-flow velocity (m h^-1^). ^∗^Values calculated based on influent concentration of 500 mg l^-1^ COD_Tot_*.

### Performance Analyses

Reactor eﬄuent was sampled on a daily basis and combined into a weekly composite sample for total COD (COD_Tot_), soluble COD (COD_Sol_), suspended COD (COD_Sus_), and colloidal COD (COD_Col_) determinations according to Standard Methods (APHA and AWWA, 2005). Protein and polysaccharide concentrations in the eﬄuent were determined by the Lowry method (Lowry et al., 1951) and the DuBois method (DuBois et al., 1956), respectively. For the measurement of total phosphorus (expressed as PO_4_^3-^) samples were passed through a 0.45 μm filter prior to analysis using the molybdovanadate Test ‘N Tube^TM^ method (Hach Lange, UK). The concentration of volatile fatty acids (VFAs) in the eﬄuent was determined by chromatographic analysis in a Varian Saturn 2000 GC/MS system (Varian Inc., Walnut Creek, CA, USA). Biogas analysis was performed by gas chromatography (Varian Inc., Walnut Creek, CA, USA) according to standard methods (APHA and AWWA, 2005).

### Biomass Characterization

#### Maximum Specific Methanogenic Activity (SMA) Testing

To evaluate changes in sludge hydrolytic and methanogenic capabilities the seed biomass and reactor biomass at HRT changes (36, 24, 12, and 8 h) were screened using the maximum specific methanogenic activity (SMA) testing method employing the pressure transducer technique as described previously (Colleran et al., 1992; Coates et al., 1996). Briefly, the test involved the measurement of the increase in biogas pressure over time following the addition of soluble substrates; propionate (30 mM), butyrate (15 mM), ethanol (30 mM), and acetate (30 mM) or of the decrease in pressure following the addition of 1 atm of H_2_/CO_2_ (80:20). Controls included vials without substrate addition and the addition of N_2_/CO_2_ (80:20) at 1 atm as a gaseous control. Tests were carried out in triplicate at 37 and 12°C. Biogas analysis was performed as described previously. Results were expressed as ml CH_4_g VSS^-1^ day^-1^.

#### Protein Degradation Assays for the Determination of *k*, *V*_max_, *A*_max_, and *K*_m_

The maximum specific activity (*A*_max_), the maximum initial velocity (*V*max), the apparent half-saturation constant (*K*_m_) and the first-order hydrolysis constant of the seed inoculum and reactor biomass were evaluated on a protein source (solubilized skimmed milk powder). These rates were determined using substrate depletion assays, which were set up similarly to the SMA test described above. Tests were performed in triplicate at 12 and 37°C using 2 g VSS l^-1^ with 2 g COD/vial of protein. The bottles were sampled at regular intervals, protein concentration was measured in the samples and a substrate depletion curve was plotted. The concentration of protein was determined using the Lowry method (Lowry et al., 1951). The kinetic parameters described above were calculated as described by Bialek et al. (2013).

#### Scanning Electron Microscopy (SEM) and Energy Dispersive X-Ray (EDX)

Scanning electron microscopy (SEM) was used to assess the structure of unused pumice stone, washed pumice stone and colonized stones and biomass from the filter, at the end of the trial. Samples were fixed by incubating in 2.5% Glutaraldehyde stock (containing 50% Glutaraldehyde, 4% Sucrose, and 0.1 M Sodium Phosphate Tribasic buffer) at 4°C overnight. Following this, a series of ethanol washes (50, 70 and 90%) were set up in individual microporous specimen cups (Canemco & Marivac, Canton de Gore, QC, Canada). Samples were then placed in each cup (50, 70, and 90, respectively) for 10 min each at room temperature. Samples were then mounted on aluminum slabs with a carbon tab (Agar Scientific, Essex, UK), incubated for 2 min at 37°C and then incubated at room temperature in sealed petri dishes with blue silica desiccants. Once the samples were dehydrated they were coated with a thin layer of gold and viewed using a SEM (Model S-4700, Hitachi, Japan). EDX was used to provide elemental composition of the samples. SEM and EDX were carried out at the National Centre for Biomedical Engineering Science (NCBES) at NUI, Galway.

#### DAPI (4′,6-Diamidino-2-Phenylindole)

4′,6-Diamidino-2-phenylindole staining was performed on granular biomass and biomass from the fixed-film filter. One hundred microliters of cells (granular biomass and free-cells suspension) were centrifuged at 18K × *g* for 10 min. The supernatant was discarded and the pellet resuspended in 100 μl of DAPI (50 μg/ml containing 150 mM KCl and 10 mM HEPES-KOH buffer pH7). The samples were incubated overnight at 4°C. The samples were then centrifuged and the pellet washed twice with reverse-osmosis water. The pellet was then resuspended in 100–200 μl of water. Ten microliters was spotted onto a slide and viewed under the microscope following air-drying. Samples were viewed using a Leica DMR microscope, a Prior L200S light source, an Olympus DP73 camera and Olympus cellSens software, with a filter cube which had an emission filter of 340–380 nm and a long-pass suppression filter of 450 nm.

### Molecular Characterization

#### DNA/RNA Co-Extraction from Biomass

Genomic DNA and RNA was extracted from granular biomass samples taken from R1 on Days 0 (Inoc), 105 (Phase 1), 209 (Phase 2), 301 (Phase 3), 361 (Phase 4A.a), 429 (Phase 4A.b), 454 (Phase 4A.c), 534 (Phase 4B.a), 596 (Phase 4B.b), and at end of the trial (Phase 5-Day 732). Biomass was sampled from the fixed-film filter at two points: – mid-trial (Day 230) and at the end of the trial (Day 732). Samples were flash frozen in liquid nitrogen and stored at –80°C prior to the extraction procedure. The nucleic acids were co-extracted by a modification of a phenol extraction method (Griffiths et al., 2000). Granular biomass (1 g) was crushed to a powder in a liquid nitrogen cooled mortar (BelArt) and 0.25 g of this powder was added into a sterile lysing matrix E tube (Fischer Scientific) prior to adding 250 μl of 1% cetyl trimethylammonium bromide (CTAB) extraction buffer, 250 μl 0.1 M Na_3_PO_4_ (pH 8) extraction buffer and 250 μl of phenol-chloroform-isoamyl alcohol (25:24:1; pH 8). Microbial cells in the samples were lysed by bead beating for 10 min at 3.2K × *g* in a Vortex-Genie2^TM^ (Scientific Industries Inc.) Phase separation was achieved by centrifugation at 13.3K × *g* for 10 min at 4°C. The clear aqueous supernatant was transferred into a sterile Phase Lock Gel^TM^ tube (Fischer Scientific) with equal volume of chloroform isoamyl alcohol (24:1). Phase separation was achieved by centrifugation at 13.3K × *g* for 10 min at 4°C. The supernatant was transferred into fresh RNase free tubes and total nucleic acids (TNA) were precipitated by using 2.5 vol of ice-cold ethanol (100%) and 1/10 vol of 3 M sodium acetate (pH 5.2) added to the extract, incubated on ice for 30 min and centrifuged (13.3K × *g*) at 4°C for 20 min. TNA were resuspended in 50 μl of diethylpyrocarbonate (DEPC) water. The integrity of each sample was assessed using agarose gel electrophoresis and quantified using a Qubit v2.0 fluorometer (Life Technologies, Darmstadt, Germany). Samples were then stored at –80°C prior to use in downstream applications. RNA was prepared by treating the TNAs with TurboDNAse (Invitrogen) according to the manufacturer’s instructions. RNA was confirmed DNA free by 16S rRNA PCR of a range of RNA dilutions. Reverse transcription was then carried out using 10 μl of DNA free RNA sample, 100 μM random hexamer primers (Invitrogen), 1 μl DEPC water following the SuperScript^TM^ III (Invitrogen) protocol according to manufacturer’s instructions. DNA and cDNA were purified with the QIAquick PCR Purification Kit (Qiagen, Germany).

#### Quantitative-Polymerase Chain Reaction (qPCR)

Quantitative-Polymerase Chain Reaction was carried out for Archaeal and Bacterial domains using DNA and cDNA generated from granular biomass and filter biomass extracted from R1. The primers 1369F and 1492R and *Taq*man probe TM1389F were used for bacterial analysis (Suzuki et al., 2000). The primers 787F and 1059R and *Taq*man probe 915F were used for archaeal analysis (Yu et al., 2005). Quantitative standard curves were constructed using standard plasmids containing the full-length 16S rRNA gene sequence from the representative bacterial strain (*Escherichia coli*) and representative archaeal strain (*Methanosarcina bakeri*). The plasmids were extracted using a Plasmid Extraction kit (BIOLINE). A PCR reaction was then carried out using the primer pairs described above. This product was cleaned using QIAQuick PCR Clean Up kit (Qiagen, Crawley, UK) according to manufacturers instructions. To construct the RT-PCR cDNA standard curves were produced from cDNA prior *in vitro* transcription of the target mRNA by using the MEGAshortscript T7 kit (Ambion) as described by Smith et al. (2006). The concentration of all standards was measured in duplicate using a Qubit system (Invitrogen) and converted into copy concentration. A 10-fold serial dilution series (10^9^–10^1^ copies ml^-1^) was generated for each standard solution and analyzed by real-time PCR, in duplicate, with its corresponding primer and probe set. The dynamic range of each standard curve was determined based on the linear regression *r*^2^ value of >0.98.

Quantitative real-time PCR was performed using a LightCycler 480 (Roche, Manheim, Germany). Each 25 μl reaction mixture was prepared using the LightCycler *Taq*Man Master Kit (Roche; 2 μl of template, 4 μl PCR-grade water, 10 μl of 2X reaction solution, 500 nM of each primer and 200 nM of probe). PCR amplification and detection was carried out as described previously (Smith et al., 2006). The volume-based concentrations (copies l^-1^) were converted to per g biomass.

#### Illumina MiSeq Analysis

Terminal Restriction Fragment Length Polymorphism (TRFLP) was used as a screening step to select samples to send for 16S amplicon sequencing. TRFLP peaks were analyzed in Peakscanner (Life Technologies^TM^). Fragments with peak height of less than 0.5% were regarded as background noise. The resulting TRFLP profiles were aligned using the web-based program T-Align with a confidence interval of 0.5 (Smith et al., 2005). The produced consensus files were then input into the software Primer 6 (PRIMER-E, Plymouth, UK) for subsequent statistical analysis. Cluster analysis, dendrograms and MDS plots of the TRFLP data were constructed using the UPGMA algorithm in Primer software beta version 6 (PRIMER-E, Plymouth, UK) after a square root transformation was applied to the matrix. This analysis allowed the identification of samples deemed interesting to aid in the selection of samples to analyze for total and active bacterial and archaeal community compositions through the construction of 16S Illumina MiSeq library generation.

From this analysis DNA and cDNA from Day 0 (Inoc), Days 105 (Phase 1), 301 (Phase 3), 454 (Phase 4A.c), 732 (End), and the filter upon take-down (FE) samples were used for 16S amplicon sequencing. The 16S rRNA gene V4 variable region was amplified using the ‘universal’ (for the co-amplification of archaeal and bacterial sequences) primer set 515F (5′-GTGCCAGCMGCCGCGGTAA-3′) and 806R (5′-GGACTACHVGGGTWTCT-AAT-3′) -Caporaso et al. (2012) with barcodes for multiplexing on the forward primer. The PCR conditions included an initial denaturation step at 94°C for 3 min, followed by 28 cycles of denaturation at 94°C for 30 s, annealing at 53°C for 40 s, and extension at 72°C for 1 min, with a final elongation step at 72°C for 5 min using HotStarTaq Plus Master Mix Kit (Qiagen, USA) for the reaction. Amplicons were then pooled in equal proportions and purified using calibrated Ampure XP beads (Beckman Coulter). The combined and purified product was prepared using the Illumina TruSeq DNA library protocol. DNA amplification and sequencing was performed at MR DNA Molecular Research Laboratory (www.mrdnalab.com; Shallowater, TX, USA) using the MiSeq reagent kit V3 (2 × 250 bp) for paired-end reads on a Solexa MiSeq machine following the manufacturer’s guidelines.

A total of 1,173489 raw 16S rRNA V4 sequences were obtained by Illumina paired end sequencing from the 12 samples. Sequence data were processed using a proprietary analysis pipeline (MR DNA, Shallowater, TX, USA; Dowd et al., 2008). This analysis involved the processing of the forward single-end read by removing barcodes, primers, sequences <200 bp, sequences with ambiguous base calls and sequences with homopolymers exceeding 6 bp. Sequences were further denoised, operational taxonomic units (OTUs) generated and chimeras removed using UCHIME (Edgar et al., 2011). 17,449 chimeric sequences were identified and removed from the samples. OTUs were defined after singleton sequences were removed, clustering at 3% divergence (97% similarity) using UCLUST (Dowd et al., 2008; Edgar, 2010). After quality processing, raw reads were reduced to 593,321 reads (Inoc DNA 70,432/cDNA 52,580, Phase 1 DNA 37, 491/cDNA 58,858, Phase 3 DNA 37,491/cDNA 21,118, Phase 4A.c DNA 37,491/cDNA 79,698, End DNA 57, 225/cDNA 43,583, FE DNA 35, 058/cDNA 49,801). Final OTUs were taxonomically classified using BLASTn against a curated GreenGenes database (DeSantis et al., 2006). In total 9,215 OTUs were identified, affiliated to 46 bacterial phyla and two archaeal classes. Raw sequences were submitted to the SRA database under the bioproject submission number PRJNA307661.

## Results and Discussion

### Phosphate Removal During AD Wastewater Treatment

Phosphate removal from the wastewater, significantly in excess of microbial growth requirements [1.5–2% of sludge dry weight (Schlegel and Zaborosch, 1993; Blackall et al., 2002)] was achieved during this trial (**Table 3**). P removal upon start-up (∼35 days) was initially high (68%) but decreased during Phase 1 (**Table 3**). P removal efficiency increased considerably during Phases 2 (to 69%) and 3 (∼78%). Following a reduction of the applied HRT to 12 h during Phase 4, P concentrations increased in the eﬄuent (**Table 3**). After the filter was changed (Phase 4B), eﬄuent P values increased with an average removal efficiency of 48%. P removal decreased further to 28% during Phase 5 corresponding to an increase in the OLR to 1.5 kg COD m^-3^ d^-1^. P removal was at its optimum during Phase 3 (OLR of 0.6 kg COD m^-3^ d^-1^).

**Table 3:** Average Phosphate concentration (in mg l^-1^) and average Phosphate, COD_Tot_, COD_Sus_, COD_Col_, COD_Sol_, Carbohydrate, Protein, and removal efficiency (RE) (%) in reactor eﬄuent and the VFA:COD ratio for the five phases of reactor operation.

Parameter	Startup	Phase 1	Phase 2	Phase 3	Phase 4A	Phase 4B	Phase 5
Phosphate conc.	13	21.5	10.5	7.6	15.2	19.5	25
RE^∗^Phosphate	61	36	69	78	55	43	28
RE^∗^COD Total	22	80	84	78	74	77	70
RE^∗^COD Suspended	0	47	60	65	37	44	36
RE^∗^COD Colloidal	17	64	37	29	13	16	0
RE^∗^COD Soluble	91	81	87	77	75	79	74
RE^∗^Carbohydrate	85	98	99	85	95	84	17
RE^∗^Protein	94	100	97	99	100	100	100
VFA:COD ratio	0.05	0.10	0.14	0.25	0.26	0.35	**–**
Theoretical CH_4_ potential^+^ (l d**^-^**^1^)	0.07	0.26	0.41	0.51	0.73	0.75	1.03

*^+^Methane potential calculated stoichiometrically, considering that all COD_totalremoved_ was converted into methane and that 1 g of COD_totalremoved_ would produce 350 ml of methane under standard temperature and pressure conditions (McCarty, 1964). The results were presented in liters per day based on the total loading of 1 day (g COD d^-1^).*

**FIGURE 1:**
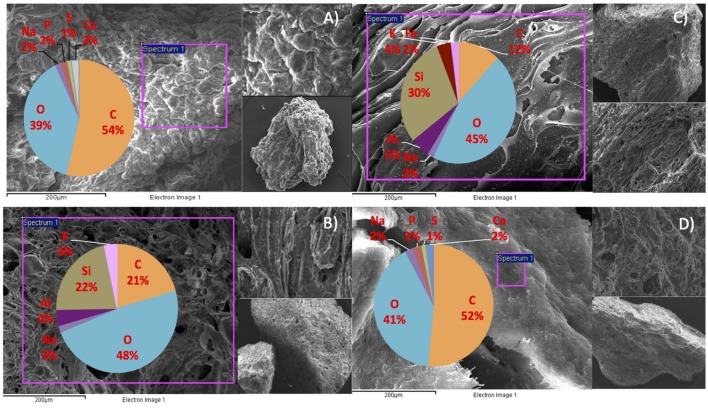
**SEM/EDX images and element composition of reactor contents at the end of the trial (A) sludge granule, (B) unused pumice stone, (C) washed pumice stone and (D) biomass on the pumice stone**.

**FIGURE 2:**
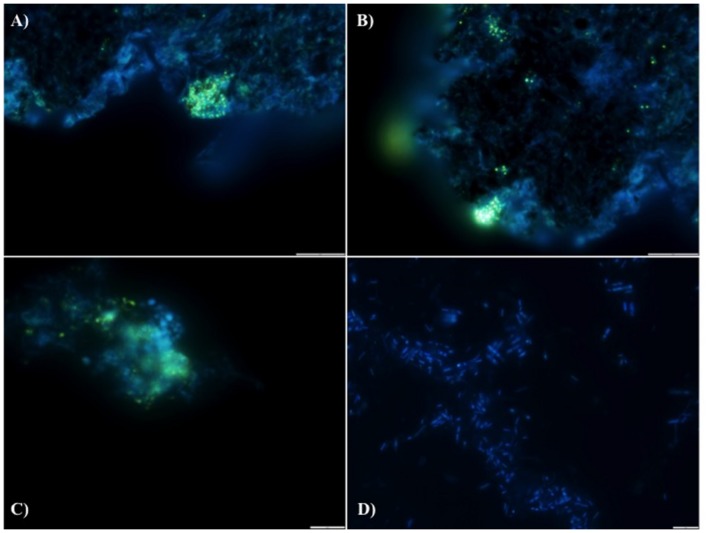
**DAPI stained images for the detection of polyphosphate (evidenced by yellow fluorescence) in reactor biomass from (A) Filter Mid-Trial, (B) Filter Mid-Trial, (C): Day 532 and (D) free cells from the filter from the end of the trial**.

**FIGURE 3:**
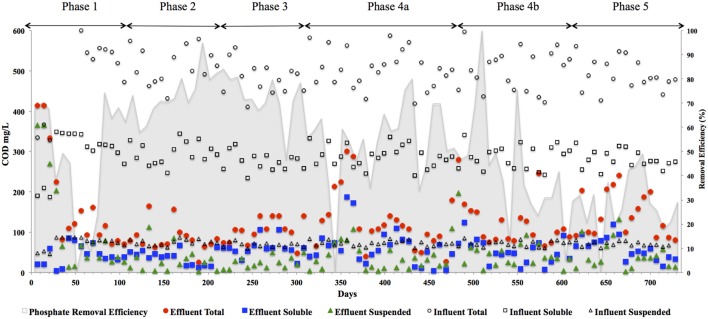
**COD_total_, COD_soluble_, COD_suspended_, and COD_colloidal_ concentrations in (mg l^-1^) in the reactor eﬄuent for the five phases of reactor operation.** Eﬄuent Total (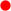), Eﬄuent Soluble (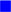), Eﬄuent Suspended (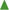), Influent Total (◯), Influent Soluble (□), Influent Suspended (△), and Influent Colloidal (◊) and phosphate removal (%) [based on an average influent concentration of 34 mg l^-1^] in shading on secondary axis.

We hypothesize that P removal in the system was biological in nature, mediated by biofilms within the reactor and the fixed-film unit rather than due to chemical precipitation (Hughes et al., 2011). Such high levels of P removal have, to date, only been associated with low-temperature AD systems (Hughes et al., 2011) and it is not known whether it could be achieved at higher temperatures or with more concentrated wastewaters. It appeared that P uptake required a period of acclimation after initial high rates during the start-up period, or perhaps a period of development associated with the colonization of biofilms within the system as evidenced by increased removal efficiency after Phase 1 (**Table 3**). It was noted that the presence of Rhodocyclus (a previously characterized PAO) had increased at this point from 0.7% in the inoculum to 4.2% in Phase 1 (based on cDNA analysis). SEM and EDX analysis carried out on sludge granules, unused pumice stone, washed pumice stone and biomass from the filter at the end of the trial demonstrated ∼2% elemental phosphorus in the biomass and colonized stones (**Figure 1**). These samples also demonstrated a 2% increase in calcium (**Figure 1**). Recent research has indicated the formation of calcium phosphate granules as a new phosphorus product during the treatment of black water (Tervahauta et al., 2014). Some calcium phosphate forms may have partially solubilized at allowing interaction with the microbial biofilm, however, calcium phosphate would be largely insoluble at lower temperatures. Most significantly, DAPI staining from biomass taken from the initial inoculum and from throughout the trial indicated biological P accumulation was occurring owing to the presence of polyP granules in the sludge bed and filter biofilms (**Figure 2**). The presence of polyP was confirmed by an enzymatic assay with ppx. PolyP was most notably observed in the biomass taken from the filter mid-trial (**Figures 2A,B**). Furthermore, phosphate removal decreased following the change of filter material (**Table 3**; **Figure 3**), this may be indicative of the loss of biomass associated with P-uptake. PolyP granules were strongly associated with large biofilm particles with free cells infrequently showing evidence of polyP. Thus, it appears that specifically, the biofilms on and within the filter materials were fundamental to P uptake. It is possible, however, that some calcium phosphate precipitation could be occurring in parallel to biological phosphorus accumulation through polyP uptake.

Pumice, the filter material employed in fixed-film section of the bioreactor, is not a new material to wastewater treatment and chemical adsorption of P in this material has also been demonstrated (Onar and Öztürk, 1993). It is the biological interaction with the material, however, which promotes efficient P removal and recovery greatly in excess of that possible through adsorption onto pumice alone (Hughes et al., 2011). It is not yet known whether the nature of the matrix material could play a role in promoting P uptake and removal via polyP formation. Indeed there is very little prior literature to provide clues as to the basis for the observed phenomenon. A study by Wang et al. (2006) described P uptake in the anaerobic phase of an EBPR reactor system. The authors hypothesized that uptake was biological in nature; but dismissed polyP accumulation as the route to P removal. The possibility of polyP uptake by anaerobic bacteria and archaea should not be readily overlooked, however. PolyP is a “key” evolutionary molecule (Kulaev and Kulakovskaya, 2000) and, as such, is found in living cells across the Bacteria and Eukarya domains (Kulaev and Kulakovskaya, 2000; Benzerara et al., 2014; Sahdeo Prasad, 2014; Kulakovskaya et al., 2015; Zhang et al., 2015). Additionally, several authors have described polyP uptake in a variety of Archaeal species (Rudnick et al., 1990; Smirnov et al., 2002; Auernik et al., 2008; Orell et al., 2012). Moreover, P removal rates continued to decrease corresponding to increases in the OLRs applied. This may indicate that carbon limitation may have been important for polyP uptake, which has been noted in known PAOs (Deinema et al., 1980). Thus, the capacity and environmental triggers for polyP formation, sufficient to facilitate biotechnological exploitation, under anaerobic methanogenic conditions warrant further investigation. Additionally, linking these parameters to the microbial populations underpinning luxury polyP uptake could provide a significant advance in biotechnological opportunities for P removal. The identification of the exact speciation of the phosphate within the system is the focus of ongoing research efforts.

In this study, we determined the biological activity profile and microbial community structure of the bioreactor system in order to provide a clearer indication of the environment in which P removal was encouraged.

### The Treatment Performance of the Hybrid Reactor

In addition to significant P removal, this system demonstrated efficient and stable process performance throughout continuous operation over 732 days at 12°C with eﬄuent COD concentrations mostly within marine discharge limits for Ireland (125 mg COD_Tot_ l^-1^; **Figure 3**). COD_Sol_ removal efficiency was stable and efficient throughout all phases of the trial. COD_Tot_ and COD_Sol_ removal efficiency values exceeded those reported in similar low-temperature sewage systems (Chu et al., 2005; Gao et al., 2011) and were comparable to results observed in an anaerobic membrane bioreactor treating domestic wastewater (Smith et al., 2013). C3–C6 VFAs were generally not detectable, while acetic acid was detected only during brief transient periods (data not shown). Eﬄuent concentrations of COD_Tot_ and COD_Sus_ were initially high (>250 mg l^-1^) due to biomass washout upon commencement of the trial, the start-up phase was short, however, lasting 35 days (**Figure 3**). Increases in the applied OLR led to transient decreases in COD removal although, in concentration terms, the increases in eﬄuent concentrations were minimal. Phase 3 was associated with a drop in the average COD_Tot_ and COD_Sol_ removal efficiencies but an increase in P removal (**Table 3**). Specific features included a decrease in the average carbohydrate removal efficiency from 99 to 85%, the appearance of acetic acid in the eﬄuent (data not shown; at low levels, up to 24 mg l^-1^) and an increased VFA:COD ratio (**Table 3**) from 0.14 (Phase 2) to 0.25 (Phase 3). These data indicated that both hydrolysis and methanogenesis were not functioning as well in Phase 3, although P removal was at it’s highest in this phase (78%). Phase 4 was divided into Phase 4A (prior to a change in the filter matrix on day 487) and Phase 4B (after the filter change). A decrease in COD_Tot_ and COD_Sus_ removal efficiencies along with evidence of filter clogging merited the replacement of the filter matrix with new pumice stone. The filter change resulted in increased overall COD removal efficiency during Phase 4B, particularly with respect to the removal of solids (**Table 3**). Particulates have been shown to comprise 85% of the COD_Tot_ in domestic sewage (Aiyuk and Verstraete, 2004; Lew et al., 2009). Our data support the idea that particulates were physically entrapped in the filter section of the reactor, allowing them to be subsequently degraded. Moreover, no accumulation of solids was observed in the granular bed section of reactor. During the fifth and final phase of operation, the average COD_Tot_, COD_Sus_, and COD_Sol_ removal efficiencies were 70, 36, and 74%, respectively (**Table 3**). The reduced performance of the reactor with respect to the degradation of particulate COD during Phase 5 indicated that a 8 h HRT was too short for complete degradation or retention of complex substrates. Another possibility for reduced performance may be related to the volumetric loading rate applied, which may not have allowed for the retention of particulates. Moreover, the sludge loading rate was much greater in this final phase than in Phase 4A (**Table 1**).

The quality of the biogas generally ranged between 50 and 60% throughout the trial. Methane yields were consistent with methanogenic activity but lower than the theoretical methane yield (**Table 3**). As no solids accumulation was observed in the system it is estimated that a large proportion of the methane generated was dissolved in the reactor eﬄuent. This is unsurprising as the recovery of methane from low-temperature systems is a known difficulty. The solubility of methane in the eﬄuent increases with decreasing temperature (Bandara et al., 2012). Several studies have demonstrated this effect. For example, Matsuura et al. (2015) found solubility increased in UASB eﬄuent by a factor of 1.4 when decreasing from 25 to 10°C, while as much as 50% of the methane generated in an AnMBR was dissolved in the reactor eﬄuent at 15°C (Smith et al., 2011). Substantial methane oversaturation been demonstrated in anaerobic eﬄuents (Hartley and Lant, 2006; Souza et al., 2011), owing to liquid-gas mass transfer limitations (Pauss et al., 1990). This is compounded further when the wastewater stream to be treated is relatively low-strength (Bandara et al., 2011; Smith et al., 2012).

### Hydrolytic Capabilities of the Biomass

The seed biomass had negligible methanogenic activity at 12°C, but increased in activity after the first phase of operation (**Table 4**). Greater activity at mesophilic temperatures, recorded throughout the trial, suggested the development of a low temperature tolerant methanogenic community, rather than a truly psychrophilic one. All assays, at both mesophilic and psychrophilic temperatures, revealed higher SMA for hydrogenotrophic methanogenesis, indicating a preference toward this route as seen previously (McHugh et al., 2006; McKeown et al., 2009; Ryan et al., 2010). The SMA on propionate at 12°C increased during the trial, indicating that an important degradation pathway was via propionate. At the end of Phase3, the SMA, at 37°C, no activity was detectable against acetate, which may explain the accumulation of acetate in the reactor eﬄuent during Phase 3. By the end of the trial, the biomass SMA on the direct methanogenic substrates H_2_/CO_2_ and acetate had increased (**Table 4**).

**Table 4:** Specific Methanogenic Activity (SMA) of reactor sludge throughout the trial at 37 and 12°C in ml Methane (CH_4_) g [VSS]**^-^**^1^ d^-1^.

	Prop^∗^	But^+^	Ethanol	Acetate	H_2_/CO_2_	*A*_max_^a^	*K*_m_^b^	*K*^c^
Seed 37°C	61^(21)^	31^(7)^	52^(17)^	80^(20)^	125^(32)^	74^(22)^	0.8^(0)^	4^(2.6)^
Seed 12°C	2^(1)^	1^(1)^	7^(2)^	3^(1)^	4^(0.2)^	35^(20)^	2.7^(0.2)^	0.6^(0)^
P 1 37°C	70^(7)^	199^(27)^	492^(82)^	272^(158)^	587^(276)^	40^(1)^	1.9^(0.1)^	4.5^(0.2)^
P 1 12°C	7^(5)^	24^(11)^	7^(6)^	12^(11)^	21^(4)^	58^(11)^	4.2^(2.5)^	1.3^(0.4)^
P 3 37°C	70^(0.5)^	273^(9)^	197^(77)^	ND	56.5^(0.4)^	67^(27)^	0.9^(0)^	0.1^(0)^
P 3 12°C	10^(13)^	10^(0.3)^	25^(3)^	ND	19^(1)^	188^(112)^	1.3^(0)^	4.2^(2.6)^
End 37°C	26^(14)^	44^(28)^	284^(135)^	176^(23)^	319^(36)^	34^(10)^	0.9^(0)^	1.7^(0.3)^
End 12°C	20^(11)^	13^(38)^	36^(32)^	9^(4)^	16^(10)^	155^(36)^	1.2^(0.1)^	3.8^(0.5)^

*^∗^Propionate; ^+^butyrate; ND, no detectable activity found. ^a^Maximum substrate utilizing rate g COD g protein^-1^d^-1^. ^b^Apparent half-saturation constant g protein l^-1^. ^c^Hydrolysis rate constant d^-1^ based on hydrolysis kinetic assays from protein depletion assays from reactor biomass throughout the trial at 37 and 12°C. Values are the mean across triplicate vials with standard deviation in brackets.*

The hybrid system degraded protein during all phases of the trial, however, with removal efficiencies between 97 and 100% (**Table 3**). This was surprising since proteins are considered to be harder to degrade than carbohydrates under anaerobic conditions, especially at cold temperatures (Aiyuk and Verstraete, 2004; Bialek et al., 2013). This result suggests that an active and efficient psychrophilic, or psychrotolerant, proteolytic group developed in the reactor. To elucidate the hydrolytic capacity of the microbial biomass, particularly with respect to protein degradation, tests using skimmed milk as a protein source were performed and various kinetic parameters (*A*_max_, *K*_m_, and *k*) were calculated. The half-saturation constant *K*_m_ was higher at 12°C than at 37°C throughout the trial (**Table 4**) indicating that microorganisms with lower substrate affinity predominated at the lower temperature, which is in agreement with a previous report (Banik et al., 1998). By the end of Phase 1, the *K*_m_ at 37 and 12°C had doubled compared to the *K*_m_ of the seed biomass, again an indication of a decrease in the substrate affinity, presumably as a response to the adaptation to cold temperatures (**Table 4**). At the end of Phase 2, the *K*_m_ decreased slightly at 37°C and decreased by 44% at 12°C suggesting that proteolytic bacteria with higher substrate affinity started to dominate in the consortium at this stage as a response to the low concentration of protein in the influent. From the end of Phase 3 until the end of the trial, the *K*_m_ decreased slightly and stabilized at both temperatures indicating further acclimatization of proteolytic bacteria with higher substrate affinity (**Table 4**).

The initial *A*_max_ (maximum specific activity; g Protein^-1^ d^-1^) was twice as large at 37°C (74 g COD g protein^-1^ d^-1^) than at 12°C (35 g protein^-1^ d^-1^), while *k* (first-order hydrolysis constant) was approximately seven times higher at 37°C than at 12°C (**Table 4**). By the end of the trial, *A*_max_ and *k* were 4.5- and 2-times higher, respectively, at 12°C than at 37°C, indicating the emergence of a psychrophilic proteolytic consortium rather than a psychrotolerant one. This is in agreement with the high protein removal levels achieved by the reactor throughout the trial.

### Molecular Characterization of the Microbial Community

#### Quantitative PCR (qPCR)

**FIGURE 4:**
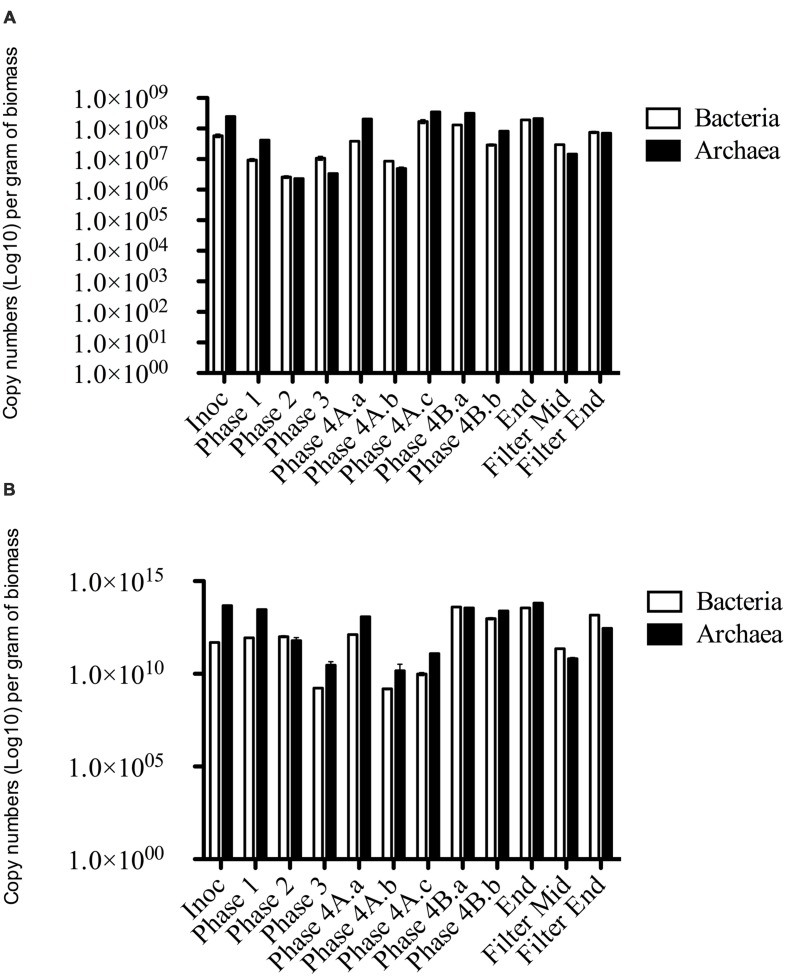
**qPCR data of Bacterial and Archaeal copy numbers (log scale) for (A) DNA and (B) cDNA from biomass samples throughout the trial**.

Archaeal gene and transcripts abundances in this study are greater than those reported by previous authors (Town et al., 2014), indicating a highly active archaeal population within this system. Temporally, archaeal transcript numbers decreased during Phase 3 (and again during Phase 4), a reduction of approximately three orders of magnitude compared to the numbers in the seed biomass. These datapoints (Phase 3, Phase 4A.a, and Phase 4A.b) immediately preceded transient deteriorations in reactor performance (**Figure 3**), and an increase in eﬄuent acetate concentrations (data not shown). The reduction also corresponded with no detectable acetoclastic activity and greatly reduced hydrogenotrophic activity in biomass sampled during Phase 3 (**Table 4**). Bacterial copy numbers also decreased at this stage (∼2 log) compared to the seed (**Figure 4**), which perhaps reflected the reduced hydrolytic capacity of the biomass sampled during Phase 3 (**Table 4**). By the end of the trial, the archaeal copy numbers were the highest recorded (6.66 × 10^13^ copies g^-1^) and the bacterial numbers had also increased by two orders of magnitude (**Figure 4**), which coincided with increases in the hydrolytic and methanogenic activity of the biomass (**Table 4**); and efficient COD removal at an OLR of 1.5 kg m^-3^ d^-1^ (**Table 3**).

#### Next Generation Sequencing

In total, 593,231 16S rRNA gene sequences >200 bp were obtained from 12 biomass samples. In total 9,215 OTUs were identified, affiliated to 46 bacterial phyla and two archaeal classes.

##### Bacterial populations

A variety of putatively fermentative and hydrolytic species were identified in the reactor. The bacterial community structure in low temperature systems has previously been indicated to be similar to that in mesophilic settings, with fermentative members of the Bacteroidetes and syntrophic members of the Proteobacteria being predominant (O’Reilly et al., 2010).

**FIGURE 5:**
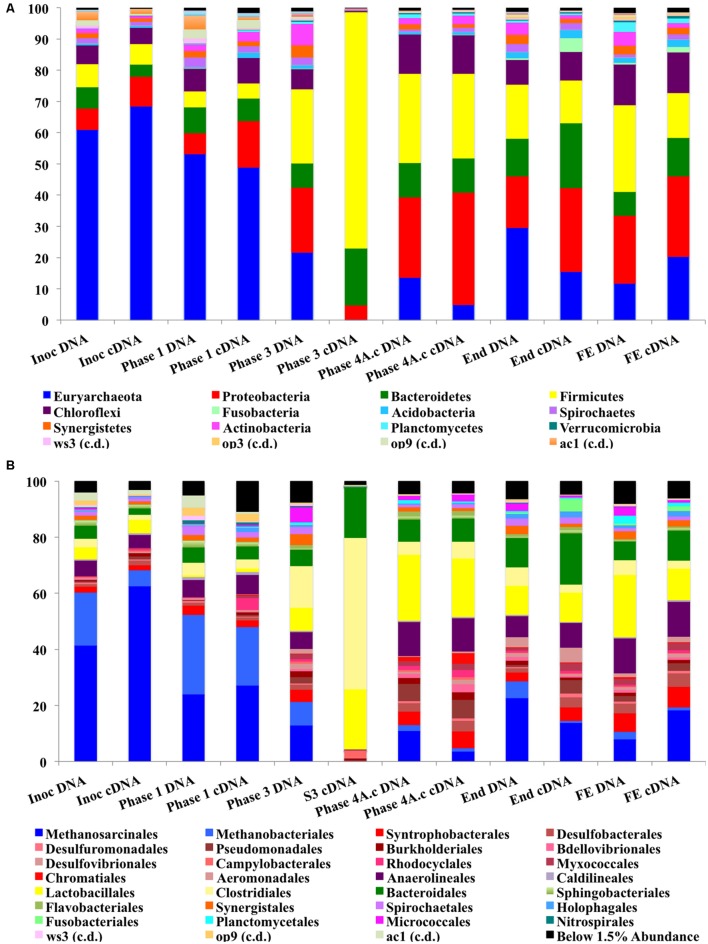
**(A)** Stacked bar charts for phylum-level Illumina MiSeq analysis. **(B)** Stacked bar charts for order-level Illumina MiSeq analysis.

The abundance of Proteobacteria increased during the trial and this was the most abundant bacterial phylum at a cDNA-level in the sludge bed and filter unit biomass, being comprised mainly of Delta, Gamma, and Beta-Proteobacteria, although Alpha and Epsilon Proteobacterial classes were also present. The phylum Proteobacteria contains a diverse consortium of species, including members isolated from low temperature environments. They are also common throughout anaerobic digestors, including mesophilic (Nelson et al., 2011) and low-temperature systems (O’Reilly et al., 2010; Bialek et al., 2012). Proteobacteria are capable of growth on a range of organic substrates (Yamada et al., 2005). Many species are associated with acetogenesis (Werner et al., 2011). The relative abundance of the phylum Firmicutes also increased dramatically during Phases 3 and 4 (**Figure 5A**) and they were the second most abundant bacterial phylum (∼27%) at the end of the trial. The most dominant classes of Firmicutes were the Bacilli and Clostridia (mainly Lactobacillales; **Figure 5B**). Psychrophilic species belonging to the class Clostridia have been isolated and identified from diverse environments (Prevost et al., 2013) and the appearance of such species in the reactor could have contributed to the development of a psychrophilic proteolytic activity, as demonstrated in the protein degradation tests (**Table 4**). The Bacteroidetes were an abundant phylum in the reactor. They increased from relatively low starting levels (ca. 10%) to reach a relatively stable level of 20–30% of the biomass. The major classes present were the Bacteroidea, Sphingobacteria, and Flavobacteria. Bacteroidetes, like the Firmicutes, play important roles in the degradation of complex organic compounds. *Flavobacterium* species have been shown to degrade protein and to possess psychrophilic proteases (Zhang et al., 2011). The Chloroflexi were also present at high levels (6–12%) throughout the trial. The *Anaerolineacea* were the dominant representative of the Chloroflexi phylum in this trial and are thought to play an important role in granulation (Yamada et al., 2005). Other phyla present included the Actinobacteria (1–7%), Fusobacteria, Acidobacteria, Caldiserica, Nitrospirae, OP8, OP9, Synergistetes, and Planctomycetes. The analysis revealed the emergence from the initial inoculum, of a small proportion of polyP-accumulating organisms (PAOs), such as *Rhodocyclus* (4% Phase 1 cDNA, negligible at the end of the trial) and *Acinetobacter* (from ∼2% from Phase 3), in granular and filter biomass.

##### Archaeal populations

The phylum Euryarchaeota represented a large majority of the sequences identified from the biomass. A clear perturbation was observed at the end of Phase 3, most notably observed in the cDNA sample (Phase 3; Day 301; **Figure 5A**). While the NGS data cannot be directly compared to the qPCR results, the qPCR data did indicate a ∼3 log reduction in archaeal 16S rRNA gene copy numbers in this phase (**Figure 4**). The perturbation coincided with the deterioration of methanogenesis in the reactor, represented by an increase in the eﬄuent VFA:COD, ratio; no detectable acetoclastic- and greatly reduced hydrogenotrophic-SMA. Furthermore, an increase in acetic acid concentrations was observed with values reaching 24 mg l^-1^ (data not shown).

The Methanosarcinales dominated the archaeal community (55–75% of the population on a DNA basis and 68–88% on a cDNA basis; **Figure 5B**). The genus *Methanosaeta* was the sole representative of the group. The dominance of *Methanosaeta* was not surprising since they have been found to dominate the methanogenic community in reactors during steady state conditions when acetate concentrations were low (Raskin et al., 1995). Although Methanosaetacea dominated the consortium in the reactor, a decrease in the relative abundance of this group was observed at the end of Phase 1 (**Figure 5B**). At the same time, the proportion of the hydrogenotrophic orders Methanobacteriales and Methanomicrobiales increased, indicating the importance of methanogenesis via H_2_/CO_2_ in the reactor. The SMA data presented earlier supports this view, as do previous reports (McKeown et al., 2009; O’Reilly et al., 2010). The increase in hydrogenotrophic methanogens and reduced acetotrophic methanogenesis, without acetate accumulation, may have been as a result of syntrophic acetate oxidation (Schnürer et al., 1996) whereby acetate is converted to hydrogen and CO_2_ by homoacetogenic bacteria. The increase in Firmicutes in the next sampling point (**Figure 5A**), which include homoacetogenic species, may be linked to the disturbance seen as both acetoclastic and hydrogenotrophic methanogens decreased. Higher eﬄuent acetate concentrations were seen at this point indicating that the homoacetogenic bacteria may have been generating acetate as the sole end product from H_2_CO_2_ or multicarbon compounds and thus, may have outcompeted the hydrogenotrophic methanogens. Homoacetogens have been reported to adapt to low temperature better than hydrogenotrophic methanogens (Kotsyurbenko et al., 2001). A drawback associated with the SMA method carried out is that this method does not account for homoacetogenic activity.

The NGS and SMA indicated a distinct disturbance to the archaeal community within the system during Phase 3. While the qPCR data supported this finding (with a decrease in archaeal numbers during Phase 3) the abundance data indicated that the archaeal community was quantitatively dominant for the majority of the trial. In fact, qPCR of Phase 3 cDNA indicated archaeal numbers were higher than bacterial numbers. qPCR has the advantage of being quantitative, specific and highly sensitive (Suzuki et al., 2000; Yu et al., 2005; Ritalahti et al., 2006). Thus, while there was a disturbance to the archaeal community they were still numerically abundant and active. There are inherent limitations to the interpretation and integration of the qPCR and sequencing data. The sequencing analysis, however, could be used to retrospectively target the microbial groups found through the development of specific primer sets.

## Conclusion

We propose here a potentially important method for sequestration of phosphorus using luxury polyP uptake under anaerobic conditions previously not described in the literature. These findings support the idea that modified AD systems could provide the basis for significant recovery and reuse of phosphorus from wastewaters, a significant advance in AD treatment technologies. However, the precise environmental and biological triggers that might promote the process of anaerobic P removal further; the exact role of the microbial biomass and the pumice filter unit; the mechanisms and conditions for anaerobic polyP formation and to what extent AD with P recovery can be developed toward a full-scale technological solution, remain to be elucidated. These questions should be a focus for on-going research efforts.

## Author Contributions

CK operated the reactor, processed all samples, generated all data, and interpreted all data. She also prepared the first draft of the manuscript and contributed to the further drafting and the finalized draft. JC and PM performed the ppx assays, the DAPI analysis on biomass throughout the trial and subsequently trained CK in the DAPI analysis. DC contributed to the design of the hydrolysis experiments and trained CK in the calculations. CS supported the design and interpretation of the microbial ecology experiments. DH designed the reactor system, contributed to the experimental design and drafting and editing of the manuscript. VOF and TM contributed to the interpretation of the reactor trial and the drafting and editing of the manuscript. JM was supervisor to the work with polyP. VO was the primary supervisor of the work.

## Conflict of Interest Statement

The authors declare that the research was conducted in the absence of any commercial or financial relationships that could be construed as a potential conflict of interest.
